# Malaria beyond its predominant endemic regions: Emerging threat or sporadic events?

**DOI:** 10.3389/fmed.2022.969271

**Published:** 2022-09-13

**Authors:** Alexandru Voloc, Joel Fleury Djoba Siawaya

**Affiliations:** ^1^Department of Pediatrics, Nicolae Testemitanu State University of Medicine and Pharmacy, Chişinău, Moldova; ^2^Centre Hospitalier Universitaire Mère-Enfant, Foundation Jeanne EBORI, Libreville, Gabon

**Keywords:** malaria, vectors, Europe, emerging threat, sporadic events

## Background

Malaria, one of the deadliest infectious diseases, has been reported to infect the Italian people since the first century (1). Although malaria was eradicated from Europe in the mid-twentieth century (1), a few cases have been reported in different regions (2, 3). In 2015, the World Health Organization (WHO) European Region reported no cases of indigenous malaria for the first time (4). The disease remained endemic in tropical regions, including sub-Saharan Africa, South East Asia, and South America (5). In 2020, the number of malaria cases was estimated at 241 million, claiming 627,000 lives (5). In the same year, Africa accounted for 96% of malaria cases and 98% of malaria-associated deaths (6). According to the WHO, countries that have had no indigenous cases for at least 3 consecutive years are considered to have eliminated malaria (5). In 2021, China and El Salvador were certified malaria-free after 4 years without malaria cases (5). Malaysia, the Islamic Republic of Iran, Belize, and Cabo Verde are on the path to malaria elimination (5).

According to the WHO, Europe is free of indigenous malaria cases (5). Malaria cases are principally imported by international travelers and immigrants (7). Data from the European Network on Surveillance of Imported Infectious Diseases revealed that “European travelers” represented 52.4% of patients with malaria in Europe between 1999 and 2000 (8). Although sustained transmission of malaria in Europe has not been identified heretofore, cases of autochthonous transmission of malaria have been reported in the past (2, 3).

## Malaria vectors

At least four potential vectors of malaria have been found in Europe. As reported in more than 20 European countries, *Anopheles atroparvus* is one of the dominant potential vectors of malaria in Europe (9). Model-based projection predicted an increase in the stability of *A. atroparvus* in southern and southeastern areas of Europe, but also intriguing was the prediction of northward spread of the vectors of *A. atroparvus* by the end of the twenty-first century (10). Next, there is *Anopheles labranchiae*, which feeds primarily on humans and is present in southern and southeastern Europe (11, 12). *Anopheles sacharovi* and *Anopheles plumbeus* are other potential vectors of malaria. The former is distributed from Italy to the Balkans, and the latter is distributed throughout Europe (12). *A. labranchiae* and *Anopheles* sacherovi also showed northward extensions in *Hertig E* predictions (10). Hertig E modeling revealed that changes in distribution were strongly governed by increasing temperature, which may be the key to the stability of malaria transmission in the region as his projection foresees [Vector Stability Index increases from 2 (1985–2005) to 8–9 in selected regions (2080–2100 forecast)] (10). The presence of malaria vectors in Europe does not necessarily translate into malaria reintroduction, even if it constitutes a condition for malaria reintroduction. This fact is clearly illustrated by the paradoxical phase of “anophelism without malaria” that followed malaria eradication from Europe.

However, recent events that have highlighted the vulnerability of these vectors to malaria parasites have raised the question of malaria resurgence. Recently, *A. labranchiae* has been implicated in the transmission of *Plasmodium malariae and Plasmodium vivax* in Europe (13–15). Additionally, there is evidence of natural infection of *A. labranchiae* by *Plasmodium falciparum* (13). *A. sacharovi* is a proven vector of malaria and has been implicated in autochthonous cases of *P. vivax* infection in Greece (16, 17). Its implication in malaria transmission has also been reported in France, Italy, Romania, and the Balkans (18). Although *A. plumbeus is* considered of minor importance in the transmission of malaria, it is an efficient carrier of malaria parasites, including *P. vivax* and *P. falciparum* (2, 7). Also, its role in malaria transmission in Europe has been documented (2, 9).

## Risk of malaria resurgence

The risk of malaria resurgence in parts of Europe depends on the combination of receptivity and vulnerability conditions. The presence of malaria vectors in Europe (principally in the countries such as France, Greece, Italy, Portugal, and Spain) and their association with the movement of population (potentially infected) from malaria-endemic regions to Europe may increase the risk of local transmission and re-emergence of malaria in Europe (3).

The 2019 EU Epidemiological Report on Malaria reported 8,641 cases of malaria, with nine confirmed cases reported as locally acquired [Germany (two cases), Greece (two cases), Spain (two cases), and France (two cases), and the Netherlands (one case)] (7). Vector-borne transmission was demonstrated in Germany and Greece (7). A study conducted in Portugal revealed that the risk of malaria resurgence exists, although it is concentrated in areas identified as hotspots (18). Romi et al. (19) assessed the risk of malaria re-emergence in central Italy using a multifactorial approach that included receptivity (the presence of local vectors and the existence of ecological and climatic conditions) and vulnerability (their presence in the country of malaria parasites capable of infesting local vectors). Their analysis suggested that the malariogenic potential of Maremma, Italy was very low. However, according to them, climatic changes toward increased temperature seem to represent a critical factor that may influence the presence, distribution, and abundance of vectors. Climatic changes we are currently experiencing, coupled with migratory flows from endemic countries, may increase the risks of malaria re-emergence in selected European countries around the Mediterranean, the Balkans, and the Black Sea.

Indeed, the high flow of travelers, the current migration crisis, and the presence of malaria vectors in countries, such as Belarus, Bulgaria, France, Greece, Italy, Portugal, Spain, and the Republic of Moldova, would increase the risk of malaria resurgence in these countries (3, 7, 11, 18–24). As a result of the sudden reduction in measures to control and prevent malaria, the risk of malaria resurgence may be particularly high in the Republic of Moldova (20). Over the years, the measures taken in the fight against the vectors of malaria have significantly decreased, leading to a significant increase in the number of mosquitoes per square meter (20, 25).

Currently, there is evidence that the triggering conditions for malaria resurgence are coming together. Entomological evidence exists as mentioned earlier. A few studies have also shown that the minimum temperatures required for *P. falciparum* and *P. vivax* to thrive are reached and run between April and October in southern Europe (22). Ongoing climatic changes can alter pathogen transmission patterns and even lead to a spatiotemporal extension of the conditions required for the development of both Plasmodium species. Moreover, thousands of cases have been reported through the years, and the source of vector infestation with Plasmodium species is present (7, 26).

## Preventing malaria resurgence in Europe

Preventing malaria resurgence in Europe requires maintaining health system awareness of the potential resurgence of malaria in Europe and planning and implementing enhanced surveillance, prevention, and control strategies (promoting awareness of preventive measures among travelers, implementing active routine vector control and malaria screening among travelers, rapid and effective diagnosis, treatment, and response).

Beyond the risk of malaria resurgence or re-emergence in Europe, the increasing number of imported malaria cases in Europe challenges us and shows that malaria is an urgent problem not only in Africa, India, Asia, Latin America, etc., but also in European countries.

## Author contributions

AV and JD conceptualized and wrote the paper. All authors contributed to the article and approved the submitted version.

## Conflict of interest

The authors declare that the research was conducted in the absence of any commercial or financial relationships that could be construed as a potential conflict of interest.

## Publisher's note

All claims expressed in this article are solely those of the authors and do not necessarily represent those of their affiliated organizations, or those of the publisher, the editors and the reviewers. Any product that may be evaluated in this article, or claim that may be made by its manufacturer, is not guaranteed or endorsed by the publisher.
